# Imaging use for low back pain by Ontario primary care clinicians: protocol for a mixed methods study – the Back ON study

**DOI:** 10.1186/s12891-019-2427-1

**Published:** 2019-02-02

**Authors:** Simon D. French, Michael E. Green, R. Sacha Bhatia, Yingwei Peng, Jill A. Hayden, Jan Hartvigsen, Noah M. Ivers, Jeremy M. Grimshaw, Christopher M. Booth, Lucia Rühland, Kathleen E. Norman

**Affiliations:** 1 School of Rehabilitation Therapy, Queen’s University, Kingston, ON Canada; 2 Department of Chiropractic, Macquarie University, Macquarie, NSW 2109 Australia; 3 Department of Family Medicine, Queen’s University, Kingston, ON Canada; 4 Department of Public Health Sciences, Queen’s University, Kingston, ON Canada; 5 Choosing Wisely Canada, Women’s College Hospital, University of Toronto, Toronto, ON Canada; 6 Institute for Health Systems Solutions and Virtual Care, Women’s College Hospital, Toronto, ON Canada; 7 Community Health and Epidemiology, Dalhousie University, Halifax, NS Canada; 8 Department of Sports Science and Clinical Biomechanics, University of Southern Denmark, Odense, Denmark; 9 Nordic Institute of Chiropractic and Clinical Biomechanics, Odense, Denmark; 10 Department of Family and Community Medicine, University of Toronto, Toronto, ON Canada; 11 Clinical Epidemiology Program, Ottawa Hospital Research Institute, Ottawa, ON Canada; 12 Department of Medicine, University of Ottawa, Ottawa, ON Canada; 13 Queen’s Cancer Research Institute, Queen’s University, Kingston, ON Canada

**Keywords:** Primary care, Low back pain, Diagnostic imaging, Cohort study, Administrative data analysis, Qualitative study

## Abstract

**Background:**

At any one time, one in every five Canadians has low back pain (LBP), and LBP is one of the most common health problems in primary care. Guidelines recommend that imaging not be routinely performed in patients presenting with LBP without signs or symptoms indicating a potential pathological cause. Yet imaging rates remain high for many patients who present without such indications. Inappropriate imaging can lead to inappropriate treatments, results in worse health outcomes and causes harm from unnecessary radiation. There is a need to understand the extent of, and factors contributing to, inappropriate imaging for LBP, and to develop effective strategies that target modifiable barriers and facilitators. The primary study objectives are to determine: 1) The rate of, and factors associated with, inappropriate lumbar spine imaging (x-ray, CT scan and MRI) for people with non-specific LBP presenting to primary care clinicians in Ontario; 2) The barriers and facilitators to reduce inappropriate imaging for LBP in primary care settings.

**Methods:**

The project will comprise an inception cohort study and a concurrent qualitative study. For the cohort study, we will recruit 175 primary care clinicians (50 each from physiotherapy and chiropractic; 75 from family medicine), and 3750 patients with a new episode of LBP who present to these clinicians. Clinicians will collect data in the clinic, and each participant will be tracked for 12 months using Ontario health administrative and self-reported data to measure diagnostic imaging use and other health outcomes. We will assess characteristics of the clinicians, patients and encounters to identify variables associated with inappropriate imaging. In the qualitative study we will conduct in-depth interviews with primary care clinicians and patients.

**Discussion:**

This will be the first Canadian study to accurately document the extent of the overuse of imaging for LBP, and the first worldwide to include data from the main healthcare professions offering primary care for people with LBP. This study will provide robust information about rates of inappropriate imaging for LBP, along with factors associated with, and an understanding of, potential reasons for inappropriate imaging.

**Electronic supplementary material:**

The online version of this article (10.1186/s12891-019-2427-1) contains supplementary material, which is available to authorized users.

## Background

Low back pain is the world’s leading cause of disability due to non-fatal health outcomes from diseases and injuries, and presents a major societal and individual burden [1, 2]. Low back pain is one of the biggest public health problems in Canada; at any one time, one in every five Canadians has low back pain [3], and it is the second most common symptom, after a cough, seen in family practice [4–6]. Each year in Canada, direct treatment costs for low back pain exceed CAD$8 billion [7], with significant additional societal costs associated with losses in worker productivity and associated disability payments.

For people with low back pain who seek care, the most common providers of choice are family practitioners, chiropractors and physiotherapists [8]. However, management of low back pain in these settings is not always concordant with recommended evidence-based practice [9–11]. Plain film x-rays are over-utilised [12, 13], and computed tomography (CT) and magnetic resonance imaging (MRI) are increasingly being utilised when not clinically indicated [11].

For patients presenting with low back pain to primary care providers, serious pathological cause is rare and up to 99% of people have “non-specific low back pain” [14]. Non-specific low back pain means pain is not attributable to a recognisable, known specific pathological cause, such as infection, tumour, fracture, or cauda equina syndrome [15]. Despite this high percentage of patients presenting with non-specific low back pain, imaging rates in the primary care setting are high [16].

Multiple international clinical practice guidelines consistently recommend that diagnostic imaging tests are not routinely indicated for people presenting with non-specific low back pain because it can lead to inefficiencies and waste in the healthcare system [17–21]. Imaging for these patients is of limited diagnostic value, provides no benefits for function, pain, or disability [22–25], may lead to worse health outcomes [26–28], and exposes people to unnecessary radiation (for x-ray and CT) [29]. Imaging may also lead to unnecessary invasive diagnostic procedures and subsequent treatment, increased waiting time for people who are in need of appropriate diagnostic imaging, poor utilisation of human resources, and excessive costs [30–33].

Despite evidence of the overuse of imaging for low back pain in other high-income countries [9, 11, 16], in Canada, there is no up-to-date information of the extent of this problem. An Ontario Institute for Clinical Evaluative Sciences (ICES) report indicated that from 1992 to 2001 the rate of CT scan and MRI use for low back pain management by family doctors in Ontario increased, along with a substantial increase in costs [34]. As well as now being out of date, this report did not determine appropriateness of imaging, nor did it examine imaging utilisation in primary care settings other than family practice. We need a better understanding of the extent of, and the factors associated with, inappropriate imaging for people with low back pain to inform effective knowledge translation interventions [35, 36].

To inform effective knowledge translation interventions relevant in Canada, we need to determine current clinician and patient beliefs. A recent systematic review of qualitative studies identified that clinicians lacked content knowledge of low back pain guidelines [37]. The few studies that have examined patient-related factors found that two out of three people had inappropriate beliefs about the use of imaging in low back pain [38–40], and most patients with low back pain expected to receive imaging [41–43]. Knowledge translation interventions are often unsuccessful because they do not adequately target the key determinants of the behaviours that need to change [44–46], whereas effective knowledge translation interventions have a strong rationale and address modifiable barriers and facilitators to the uptake of evidence-based care [47]. Thus, there is mounting evidence suggesting that the design of knowledge translation interventions requires a more systematic approach with a strong rationale for the chosen design and explicit reporting of the intervention development process [48–50].

We need a better estimate of the extent of inappropriate imaging for low back pain in Canadian primary care. We also need to obtain reliable information to inform the design of effective knowledge translation interventions to improve the quality of low back pain care. This project will provide new knowledge to inform the design of tailored strategies to reduce inappropriate imaging for low back pain. These interventions will have the potential to improve the use of healthcare resources and reduce harm to the vast number of people who seek care for low back pain in Canada.

## Methods

Our overall project goal is to inform the design of evidence-based and theoretically-informed knowledge translation strategies to reduce inappropriate imaging for people with low back pain in Ontario, targeting primary care clinicians, patients and the public. To achieve this goal, we will undertake a mixed methods study incorporating an inception cohort study (Study 1) and a qualitative study of primary care clinicians and patients (Study 2).

### Study objectives

#### Primary objectives

1. To determine the rate of, and factors associated with, inappropriate lumbar spine imaging (x-ray, CT scan and MRI) for people with non-specific low back pain presenting to primary care clinicians in Ontario.
2. To determine the barriers and facilitators to reducing inappropriate imaging for low back pain in primary care settings.

#### Secondary objectives

3. To determine differences in rates of recommending imaging among types of primary care clinicians.
4. To describe demographic characteristics and patterns of care for people with low back pain in primary care settings.

### Methods for study 1

#### Clinician and patient recruitment

*Clinician participants (n = 175)* will comprise 50 each from chiropractic and physiotherapy, and 75 from family medicine. We are recruiting more family medicine clinicians than the other professions because lower numbers of patients with low back pain attend family medicine than chiropractic and physiotherapy. Our partner organisations will promote study participation via their normal communication strategies to their clinician members. We will invite clinicians in community-based practices chosen randomly from the lists of all registered clinicians in Ontario. From the College of Physicians and Surgeons of Ontario we will select only family doctors. From the College of Physiotherapists of Ontario we will select only physiotherapists whose primary work site is primary care. From the College of Chiropractors of Ontario we will use the full list.

We anticipate a 30% response rate based on our previous experience [51, 52], hence we will approach at least 600 clinicians to reach our sample size. To ensure the efficient use of project resources, we will target clinicians who practice in seven geographic regions within Ontario, Canada. These regions were selected to be representative of the mix of high and low population density areas, and different geographical regions: 1) Sudbury/North Bay; 2) Mississauga/Halton (Mississauga; Oakville; Milton); 3) Kitchener, Waterloo, and Guelph; 4) London, Ontario; 5) Central Toronto; 6) Southeast Ontario (Belleville; Brockville; Napanee; Smiths Falls; Kingston; Picton; Gananoque; Perth); 7) Ottawa region (Ottawa; Kanata; Nepean; Orleans).

We will invite clinicians to participate using a modified tailored design method [53] by sending a primer postcard, an invitation letter, two reminder letters and a telephone call. We will also use a snowball sampling technique, where, after clinicians are recruited, they will be asked to approach clinicians who work in the same practice, or other clinicians they know who they believe may be interested in participating.

Recruited clinicians will complete a baseline demographic questionnaire including: sex; age; training location; years in practice; and proximity of practice to imaging facility (see Additional file 1: Appendix 1 for questionnaire). Results of this questionnaire will be used to assess clinician representativeness by comparing to available province-wide clinician demographic characteristics and also be used as clinician-related factors when modeling inappropriate use of imaging.

*Patient participants (n = 3750):* Consecutive patients with low back pain will be invited to participate until up to 25 are recruited from each participating clinician. Research staff will visit each participating practice to train the practice staff and clinicians in the study procedures. To reduce possible selection bias, we will encourage practice staff at each location to follow a strategy suitable for their practice that maximises the chances of recruiting consecutive patients with low back pain. For clinicians in solo practice without reception staff, typical of some chiropractors and physiotherapists, clinicians will approach potential participants directly. For clinicians in circumstances where practice reception staff are likely to know in advance that a patient’s consultation is for low back pain, practice reception staff will be asked to inform every such patient about the study during the recruitment period and provide them with recruitment information to read in the waiting room. If the patient consents to participate, the practice staff will inform the clinician, who will then complete the enrolment process, including screening for participation.

We are allowing 12 months for patient recruitment; that is, approximately two patients with low back pain recruited per month per clinician, which is feasible based on incidence rates of low back pain presentations in these settings [4, 54, 55]. Practices will be offered reimbursement for staff time required to recruit patients and collect data.

Rates of patient recruitment will be monitored via contact from the research team to clinicians and practice staff approximately every 2 weeks. We will ask participating clinicians to keep a de-identified list of patients whom they approached, and to indicate how many were ineligible and how many declined participation. To reduce the risk of selection bias, practice staff, clinicians and patients will only be told that this is an observational study on the current management of low back pain in primary care, and not that lumbar imaging is the main focus. There is a lower incidence of low back pain presentations in family medicine compared to chiropractic and physiotherapy, so patient recruitment in this setting will require more support [56].

#### Inclusion/exclusion criteria

*Clinician participants* will be currently practising and providing care for patients with low back pain. Their practice must be a first point of contact for ambulatory care in Ontario, and they must be in good standing with their respective Ontario regulatory body. We will exclude family doctors who practice in academic family health teams due to the presence of residents rotating through these practices.

*Patient participants* will be eligible if they are consulting participating clinicians for the first time for an episode of low back pain, with or without leg pain, and they have an Ontario Health Insurance Plan (OHIP) number. Patients will be included if they are aged ≥18 years old with a new episode, or acute exacerbation, of low back pain. This is defined as the current episode of pain preceded by at least four weeks without significant low back pain; we define significant low back pain when average pain intensity is scored as 3 or more on a scale of 0 to 10, and the pain interferes with daily activities. Pain will be in the region bound by the lower ribs and the lower gluteal fold, with or without pain referred beyond this region. Patients will be excluded if they are pregnant, or if they are unable to speak and read English. Where possible, practice staff will use a checklist to screen potential patients for eligibility (see Additional file 1: Appendix 2). Otherwise, participating clinicians will screen patients for inclusion at baseline.

#### Data collection and data sources

Table 1 and Table 2 describes the patient-level and clinician-level factors that we hypothesise may be associated with inappropriate imaging.

**Table 1:** Patient-level factors being measured that may be associated with inappropriate imaging

Factor	Questionnaire items	Timing				
		*First visit*	*Follow up visits*	*3-month Q*	*6-month Q*	*12-month Q*
Age	Single questionnaire item	X				
Sex	Single questionnaire item	X				
Education level	Single questionnaire item	X				
Socio-economic status	Two questionnaire items: Postal code and education level	X				
Race/ethnicity	Single questionnaire item	X				
Living arrangement	Single questionnaire item	X				
Insurance status	Single questionnaire item	X				
Compensation status (workplace injury or motor vehicle)	Single questionnaire item	X				
Patient expectations of imaging	Single questionnaire item	X	X			
Patient beliefs about imaging	Two questionnaire items [43]	X				
Duration of low back pain	Single questionnaire item	X				
Previous history of low back pain	Single questionnaire item	X				
Low back pain intensity	Single questionnaire item (pain right now)	X	X	X	X	X
Leg pain intensity	Single questionnaire item (pain right now)	X	X	X	X	X
Recovery expectations	Single questionnaire item	X	X			
Previous back surgery	Single questionnaire item	X				
Previous medication and non-medication treatment	Single questionnaire items	X				
Previous imaging	Single questionnaire item	X				
General Health	Single questionnaire item	X	X			
Physical activity level	Single questionnaire item	X				
Overweight or obesity	Two questionnaire items: Self-reported height and weight	X				
Possible pathological cause of back pain (fracture, infection, cancer, cauda equina syndrome)	Questionnaire items about: Trauma; History of cancer; Unexplained weight loss; IV drug user; Long term use of oral steroids	X	X	X	X	X
Anxiety	Two questionnaire items [73]	X	X			
Depression	Two questionnaire items [73]	X	X			
Confidence in ability to work and to live a normal lifestyle	Two questionnaire items	X	X			
Disability	Questionnaire items: Roland Morris Q [74]	X		X	X	X
STarT Back risk category	Questionnaire items: STarT Back questionnaire [75, 76]	X		X	X	X
Fear avoidance	Single questionnaire item 14 from the Tampa Scale for Kinesiophobia (TSK) [77]^a^	X	X			
Pain Catastrophizing	Single questionnaire item 3 from Pain Catastrophizing Scale (PCS) [78]^a^	X	X			
Patient satisfaction	Single questionnaire item of global perceived effect		X			
Adverse events	Single questionnaire item [79]		X			

^a^Asked as part of STarT Back questionnaire at first visit

**Table 2:** Clinician-level factors predictive of inappropriate imaging being investigated, all measured via baseline clinician questionnaire

Factor	Questionnaire items
Age	Single questionnaire item
Sex	Single questionnaire item
Years in practice	Single questionnaire item
Clinician type (family doctor, chiropractor, physiotherapist)	Single questionnaire item
School of entry-to-practice training (for everyone); program of family medicine training (for family doctors only)	Single questionnaire item
Access to imaging (onsite or offsite)	Single questionnaire item asked as part of a list of items about onsite services
Ownership of imaging facilities	Single questionnaire item
Self-identification as having a speciality in back pain	Single questionnaire item [80]
Clinician fear avoidance beliefs	Single questionnaire item: 14 from the Tampa Scale for Kinesiophobia (TSK), modified for clinician [81]
Average time spent with patient	Single questionnaire item
Type of manual technique modalities practiced (for chiropractors)	Single questionnaire item
Beliefs on usefulness of imaging	Single item on questionnaire
Knowledge of imaging guidelines	Single item on questionnaire

### Encounter data

After each patient consultation during the data collection period, participating clinicians will complete a handwritten 1-page encounter form (see Additional file 1: Appendix 3) for each of 25 consecutive consenting eligible patients with low back pain. There is one type of form for the initial encounter, and another, briefer form, for any follow-up consultations for the same low back pain episode. The encounter form is a modified form previously used in a chiropractic setting to systematically collect clinical data [51]; chiropractors reported that completing the form took 2–3 min per patient. The encounter forms for this project were piloted by two chiropractors, three physiotherapists and two family practitioners, who each recruited between two to five patients with low back pain over a six week period. Only minor changes were made to the form after the pilot study.

At the time of the patient’s initial consultation clinicians will record: key history and clinical findings; working diagnosis; diagnostic imaging ordered/taken; interventions provided or recommended; who is paying for the consultation; and, OHIP number for linkage purposes. Follow up encounter forms will be completed for each visit for up to 6 weeks to determine if any clinical indications for imaging arise. Six weeks was chosen because this is the time when people with low back pain are likely to be recovered, if pain is persisting they have progressed to the sub-acute stage, or if they have a pathological cause this is likely to be definitively diagnosed [57].

### Patient questionnaires

**Fig. 1:**
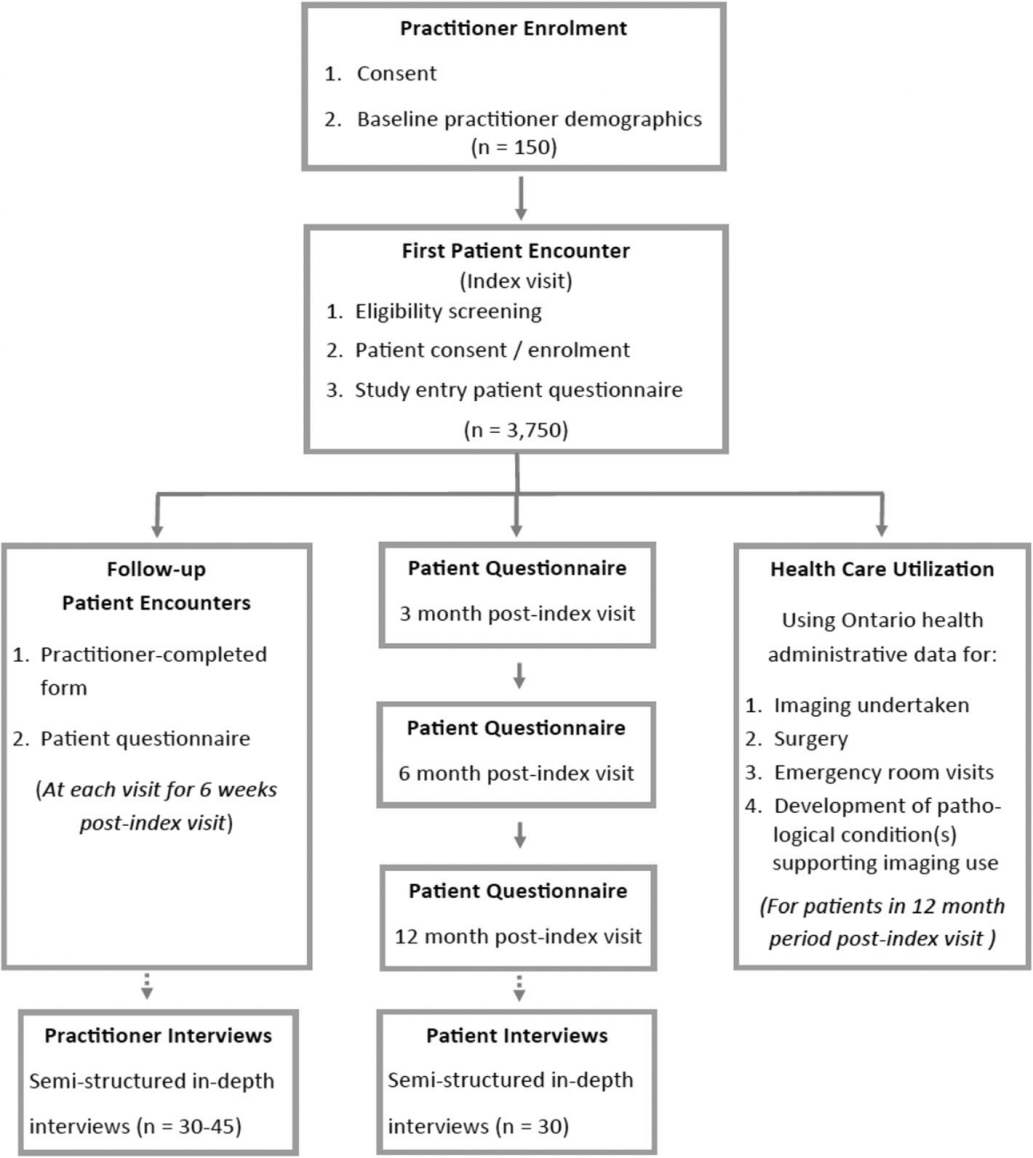
Flow chart of participant recruitment and data collection

Patients will complete questionnaires at each visit for up to six weeks (Table 1 provides domains covered, and the Additional File (Appendices 4 and 5) includes the questionnaires). Patients will also complete online questionnaires at 3 months, 6 months and 12 months post-index visit (see Additional file 1: Appendix 6). Figure 1 documents the patient data collection time points throughout the study.

### Administrative data

Participating patients will be followed for 12 months using Ontario health administrative data held by the Ontario Institute for Clinical Evaluative Sciences (ICES) to determine if any imaging was undertaken (plain x-ray, CT scan, MRI, fluoroscopy, positron emission tomography (PET) scan, ultrasound). Although plain x-ray, CT scan and/or MRI are most likely to be ordered for patients with low back pain, we will capture all related imaging to be comprehensive in the assessment of inappropriate imaging. ICES data will also capture whether a pathological condition develops (defined with diagnoses at more than two healthcare visits), and surgery and emergency visits related to such a condition.

Some imaging may not be captured by the administrative data. Approximately 12% of chiropractors take their own plain x-rays and this information will not be captured in ICES data [58]; completed encounter forms by these chiropractors will indicate whether they took lumbar x-rays, and this information will also be collected via the patient-completed questionnaires. Also, some patients in Ontario elect to go to a private facility outside Ontario and individually pay for imaging. This information will be captured via the patient-completed questionnaires.

### Inappropriate imaging

The encounter, questionnaire and administrative data will be used to determine if any imaging ordered is inappropriate. We will apply a modified version of the American College of Radiology imaging appropriateness criteria for low back pain [59]. If any of the following situations occur, including signs or symptoms indicating suspicion of “red flag” conditions [60], imaging will be considered appropriate: infection, tumour, fracture, or cauda equina syndrome (Table 3). If the clinical, questionnaire and administrative data are not suggestive of any of these conditions, any lumbar imaging ordered will be deemed inappropriate.

**Table 3:** Diagnostic imaging will be deemed inappropriate in the absence of these conditions that indicate a specific cause of low back pain [59]

Condition	Sign or symptom
Cancer	History of cancer; unexplained weight loss
Infection	Immunosuppression; intravenous drug use
Fracture	Prolonged use of corticosteroids; history of significant trauma; minor fall or heavy lift in a potentially osteoporotic or elderly individual
Cauda equina syndrome	Acute onset of urinary retention or overflow incontinence; faecal incontinence; saddle anaesthesia

### Data entry and coding

Trained secondary coders will undertake independent double data entry of encounter forms into the study’s purpose built database. Encounter data will be coded using the International Classification of Primary Care (ICPC-2), adopted by the World Health Organization as a classification system for primary care [61, 62], and for the chiropractic data ICPC-2 PLUS CHIRO [63]. A quality assurance protocol to ensure reliability of data entry, including the development of computer-aided error checks (‘locks’) at the data entry stage, will be built into the database. Further range and logical data checks will be conducted within the database.

### Data access plan

ICES, funded by the Ontario Ministry of Health and Long Term Care, links encoded population-based health information at the patient level. The practice-level and patient questionnaire data collected by the study team will be migrated to ICES for linkage. Analysis will be conducted using the following ICES datasets: OHIP database for imaging fee codes; OHIP Claims Database; National Ambulatory Care Reporting System Database for emergency room visits; Canadian Institute for Health Information Database for hospitalisation and surgery; Ontario Cancer Registry.

### Sample size justification

The study will be adequately powered to determine factors associated with imaging behaviour, including both clinician (e.g. profession) and patient (e.g. severity and duration of pain) characteristics. We will compare inappropriate imaging rates among professions, assuming base imaging rates across the professions range from 10 to 45% [9, 11, 34, 64, 65]. Using an intracluster correlation coefficient estimate of 0.088 [66, 67], and 25 as the cluster size (3750 total patients), this study will have at least 95% power to detect an absolute difference in imaging rates between professions of 6%. For patient factors, we hypothesise that increased pain severity and increased pain duration may be associated with inappropriate imaging [43], this study will have at least 90% power to detect a 10% difference in imaging rates with each 2-point increase in pain severity measured by the numerical rating scale (0 to 10), or each 2-week increased duration of low back pain.

### Quantitative data analyses

Characteristics of clinicians, patients and encounters will be summarised using descriptive statistics. We will calculate imaging rates per 100 low back pain patients with 95% confidence intervals and we will determine imaging rates for each primary care profession. We will undertake multilevel logistic regression analysis to determine the effects of clinician- and patient-level factors on inappropriate imaging. Clinician-level factors assessed will include age, sex, profession type, location of clinician training, clinician years in practice, ownership of imaging facilities, knowledge of imaging guidelines, and time spent with patient. Patient-level factors measured at baseline will include age, sex, race/ethnicity, insurance status, living in a metropolitan area, beliefs about usefulness of imaging, education level, duration/severity of low back pain, and three month measurement of satisfaction with treatment and adverse events. Patients consulting each clinician will form a cluster and will be considered as the second level in the multilevel logistic regression. We will also conduct a marginal logistic regression analysis to investigate the effects of clinician- and patient-level factors on inappropriate imaging and estimate the association between inappropriate imaging and explanatory factors in the marginal model using Generalised Estimating Equations. The analysis will allow examination of differences in results from the two models and determine whether the conclusions depend unduly on the model assumptions.

### Methods for study 2

#### Procedure

We will conduct semi-structured in-depth interviews with clinicians from each profession and their patients. Study 1 clinician and patient participants will be eligible to participate. We will purposefully approach individuals to provide a variety of views and perspectives, including patients who have, and who have not, been referred for imaging. Patients will be approached for an interview during the early stages of their management for low back pain so that their experience is recent. Clinicians will not be interviewed until after they complete their participation in Study 1 to avoid revealing the true nature of the study to them.

Our target sample size will be 30–45 clinicians (10–15 from each profession) and 30 patients (10 who consult each profession). Recruitment and sampling will cease once our data analysis has produced a stable set of findings and emergent codes no longer arise during data collection and analysis (i.e. once we have reached theoretical saturation) [68].

Semi-structured interviews will be conducted over the telephone by a project staff member. Interview questions will be informed by the Theoretical Domains Framework (TDF) to systematically identify barriers and facilitators to practising in accordance with best practice recommendations, focussing on the use of inappropriate imaging [69, 70]. Interviews will be audio-recorded and transcribed verbatim.

For clinician interviews, we will ask general questions about how they manage patients with a new episode of low back pain. We will ask about their knowledge of low back pain evidence-based guidelines to gain insight into the factors that hinder, or facilitate, clinical behaviour that is in accordance with the guidelines [17]. For clinicians who order imaging, we will also ask about what benefits they feel they get from imaging when they refer for it, if they feel it changes their patient management, and how they use imaging information. The interview guide will be piloted with two clinicians from each profession prior to data collection to assess its comprehensiveness, practicability and acceptability. For patient interviews we will ask about their expectations and beliefs regarding the need for imaging for their low back pain, as well as more general questions about their beliefs and attitudes about low back pain, generated from items from the Back Pain Beliefs Questionnaire [71].

### Qualitative data analysis

We will undertake content and thematic analysis of interview transcripts and both the manifest and latent content will be examined [72]. Both clinician and patient datasets will be categorised in relation to consistency with guideline recommendations for imaging. Where possible, we will also explore the perceptions of the patient and clinician as dyads to understand how this interaction may influence imaging decisions. Data will be analysed to identify barriers and facilitators that influence clinicians’ clinical behaviour about imaging for low back pain. Factors will be thematically mapped to the domains of the TDF. Factors emerging on multiple occasions, or considered likely to influence clinical behaviour, will be deemed relevant to identify important theoretical domains. The initial analysis of interview data will be undertaken by a project staff member (with qualitative method expertise), and a random subset of 20% of interviews will be independently coded and analysed by a second researcher (research assistant or graduate student) as verification of the initial analysis. Concordance between coders will be determined and consensus reached. When discrepancies remain, these will be discussed with a senior investigator until agreement is reached.

### Project advisory committee

Our project Advisory Committee comprises all investigators and representatives of the partner organisations. Partner organisations include the Ontario Physiotherapy Association, the Ontario Chiropractic Association and the Ontario College of Family Physicians. The committee meets at least twice each year over the project duration to advise on all aspects of the project.

## Discussion

This will be the first Canadian study to document the extent of the overuse of imaging for low back pain, and the first worldwide to include data from all relevant professions offering primary care for people with low back pain and who can refer for imaging. The outputs will be robust information about rates of inappropriate imaging for low back pain, along with predictors and reasons why inappropriate imaging is occurring. The study will also reveal differences in rates of recommending imaging among types of clinicians, and describe demographics and patterns of care for people with low back pain in primary care settings. Using both quantitative and qualitative results, knowledge translation strategies will be developed to address the issue of inappropriate imaging in these professions. Working with our project partners, the knowledge translation strategies will aim to be relevant, feasible and acceptable for each profession and the general public.

## Additional file

Additional file 1: Data collection forms. (DOCX 331 kb)

## Abbreviations

CT: Computed tomography
ICES: Institute for Clinical Evaluative Sciences
ICPC-2: International Classification of Primary Care
LBP: Low back pain
MRI: Magnetic resonance imaging
OHIP: Ontario Health Insurance Plan
PET: Positron emission tomography
TDF: Theoretical Domains Framework

## References

[1] Vos T, Flaxman AD, Naghavi M, Lozano R, Michaud C, Ezzati M (2012). Years lived with disability (YLDs) for 1160 sequelae of 289 diseases and injuries 1990-2010: a systematic analysis for the global burden of disease study 2010. Lancet.

[2] Hartvigsen J, Hancock MJ, Kongsted A, Louw Q, Ferreira ML, Genevay S (2018). What low back pain is and why we need to pay attention. Lancet.

[3] Lim KL, Jacobs P, Klarenbach S (2006). A population-based analysis of healthcare utilization of persons with back disorders: results from the Canadian community health survey 2000-2001. Spine (Phila Pa 1976).

[4] Rapoport J, Jacobs P, Bell NR, Klarenbach S (2004). Refining the measurement of the economic burden of chronic diseases in Canada. Chronic Dis Can.

[5] Wandell P, Carlsson AC, Wettermark B, Lord G, Cars T, Ljunggren G (2013). Most common diseases diagnosed in primary care in Stockholm, Sweden, in 2011. Fam Pract.

[6] Britt H, Miller G, Henderson J, Bayram C, Harrison C, Valenti L (2015). General practice activity in Australia 2014–15. General practice series no. 38.

[7] Coyte PC, Asche CV, Croxford R, Chan B (1998). The economic cost of musculoskeletal disorders in Canada. Arthritis Care Res.

[8] Walker BF, Muller R, Grant WD (2004). Low back pain in Australian adults: health provider utilization and care seeking. J Manip Physiol Ther.

[9] Williams CM, Maher CG, Hancock MJ, McAuley JH, McLachlan AJ, Britt H (2010). Low back pain and best practice care: a survey of general practice physicians. Arch Intern Med.

[10] Runciman WB, Hunt TD, Hannaford NA, Hibbert PD, Westbrook JI, Coiera EW (2012). CareTrack: assessing the appropriateness of health care delivery in Australia. Med J Aust.

[11] Mafi JN, McCarthy EP, Davis RB, Landon BE (2013). Worsening trends in the management and treatment of back pain. JAMA Intern Med.

[12] Good Stewardship Working Group (2011). The "top 5" lists in primary care: meeting the responsibility of professionalism. Arch Intern Med.

[13] Somerville S, Hay E, Lewis M, Barber J, van der Windt D, Hill J (2008). Content and outcome of usual primary care for back pain: a systematic review. Br J Gen Pract.

[14] Henschke N, Maher CG, Refshauge KM, Herbert RD, Cumming RG, Bleasel J (2009). Prevalence of and screening for serious spinal pathology in patients presenting to primary care settings with acute low back pain. Arthritis Rheum.

[15] Balague F, Mannion AF, Pellise F, Cedraschi C (2011). Non-specific low back pain. Lancet.

[16] Jenkins HJ, Downie AS, Maher CG, Moloney NA, Magnussen JS, Hancock MJ. Imaging for low back pain: is clinical use consistent with guidelines? A systematic review and meta-analysis. Spine J. 2018. 10.1016/j.spinee.2018.05.004 29730460

[17] Koes BW, van Tulder M, Lin CW, Macedo LG, McAuley J, Maher C (2010). An updated overview of clinical guidelines for the management of non-specific low back pain in primary care. Eur Spine J.

[18] Institute of Health Economics (IHE) (2011). Guideline for the evidence-informed primary care management of low back pain.

[19] Traeger A, Buchbinder R, Harris I, Maher C (2017). Diagnosis and management of low-back pain in primary care. CMAJ.

[20] Almeida M, Saragiotto B, Richards B, Maher CG (2018). Primary care management of non-specific low back pain: key messages from recent clinical guidelines. Med J Aust.

[21] Oliveira CB, Maher CG, Pinto RZ, Traeger AC, Lin CC, Chenot JF (2018). Clinical practice guidelines for the management of non-specific low back pain in primary care: an updated overview. Eur Spine J.

[22] Chou R, Fu R, Carrino JA, Deyo RA (2009). Imaging strategies for low-back pain: systematic review and meta-analysis. Lancet.

[23] Gilbert FJ, Grant AM, Gillan MGC, Vale L, Scott NW, Campbell MK (2004). Does early imaging influence management and improve outcome in patients with low back pain? A pragmatic randomised controlled trial. Health Technol Assess.

[24] Kendrick D, Fielding K, Bentley E, Miller P, Kerslake R, Pringle M (2001). The role of radiography in primary care patients with low back pain of at least 6 weeks duration: a randomised (unblinded) controlled trial. Health Technol Assess.

[25] Kerry S, Hilton S, Dundas D, Rink E, Oakeshott P (2002). Radiography for low back pain: a randomised controlled trial and observational study in primary care. Br J Gen Pract.

[26] Ash LM, Modic MT, Obuchowski NA, Ross JS, Brant-Zawadzki MN, Grooff PN (2008). Effects of diagnostic information, per se, on patient outcomes in acute radiculopathy and low back pain. AJNR Am J Neuroradiol.

[27] Webster BS, Bauer AZ, Choi Y, Cifuentes M, Pransky GS (2013). Iatrogenic consequences of early magnetic resonance imaging in acute, work-related, disabling low back pain. Spine (Phila Pa 1976).

[28] Webster BS, Choi Y, Bauer AZ, Cifuentes M, Pransky G (2014). The cascade of medical services and associated longitudinal costs due to nonadherent magnetic resonance imaging for low back pain. Spine (Phila Pa 1976).

[29] de Gonzalez AB, Darby S (2004). Risk of cancer from diagnostic X-rays: estimates for the UK and 14 other countries. Lancet.

[30] Lurie JD, Birkmeyer NJ, Weinstein JN (2003). Rates of advanced spinal imaging and spine surgery. Spine (Phila Pa 1976).

[31] Deyo RA, Mirza SK, Turner JA, Martin BI (2009). Overtreating chronic back pain: time to back off?. J Am Board Fam Med.

[32] Deyo RA (2002). Cascade effects of medical technology. Annu Rev Public Health.

[33] Chou R, Deyo RA, Jarvik JG (2012). Appropriate use of lumbar imaging for evaluation of low back pain. Radiol Clin N Am.

[34] Iron K, Jaakkimainen L, Rothwell DM, Ping L, Laupacis A (2004). Investigation of acute lower back pain in Ontario: are guidelines being followed?.

[35] Jenkins HJ, Hancock MJ, French SD, Maher CG, Engel RM, Magnussen JS (2015). Effectiveness of interventions designed to reduce the use of imaging for low-back pain: a systematic review. CMAJ.

[36] French SD, Green S, Buchbinder R, Barnes H (2010). Interventions for improving the appropriate use of imaging in people with musculoskeletal conditions. Cochrane Database Syst Rev.

[37] Slade SC, Kent P, Patel S, Bucknall T, Buchbinder R (2015). Barriers to primary care clinician adherence to clinical guidelines for the management of low back pain: a systematic review and meta-synthesis of qualitative studies. Clin J Pain.

[38] Espeland A, Baerheim A, Albrektsen G, Korsbrekke K, Larsen JL (2001). Patients' views on importance and usefulness of plain radiography for low back pain. Spine.

[39] Werner EL, Ihlebaek C, Laerum E, Wormgoor ME, Indahl A (2008). Low back pain media campaign: no effect on sickness behaviour. Patient Educ Couns.

[40] Moffett JAK, Newbronner E, Waddell G, Croucher K, Spear S (2000). Public perceptions about low back pain and its management: a gap between expectations and reality?. Health Expect.

[41] Stafford V, Greenhalgh S, Davidson I (2014). Why do patients with simple mechanical back pain seek urgent care?. Physiotherapy.

[42] Hoffmann TC, Del Mar CB, Strong J, Mai J (2013). Patients' expectations of acute low back pain management: implications for evidence uptake. BMC Fam Pract.

[43] Jenkins HJ, Hancock MJ, Maher CG, French SD, Magnussen JS (2016). Understanding patient beliefs regarding the use of imaging in the management of low back pain. Eur J Pain.

[44] French SD, Green SE, O'Connor DA, McKenzie JE, Francis JJ, Michie S (2012). Developing theory-informed behaviour change interventions to implement evidence into practice: a systematic approach using the theoretical domains framework. Implement Sci.

[45] Davies P, Walker AE, Grimshaw JM (2010). A systematic review of the use of theory in the design of guideline dissemination and implementation strategies and interpretation of the results of rigorous evaluations. Implement Sci.

[46] Grimshaw JM, Thomas RE, MacLennan G, Fraser C, Ramsay CR, Vale L (2004). Effectiveness and efficiency of guideline dissemination and implementation strategies. Health Technol Assess.

[47] Grol RP, Bosch MC, Hulscher ME, Eccles MP, Wensing M (2007). Planning and studying improvement in patient care: the use of theoretical perspectives. Milbank Q.

[48] Des Jarlais DC, Lyles C, Crepaz N (2004). Improving the reporting quality of nonrandomized evaluations of behavioral and public health interventions: the TREND statement. Am J Public Health.

[49] Baker EA, Brennan Ramirez LK, Claus JM, Land G (2008). Translating and disseminating research- and practice-based criteria to support evidence-based intervention planning. J Public Health Manag Pract.

[50] Boutron I, Moher D, Altman DG, Schulz KF, Ravaud P (2008). Extending the CONSORT statement to randomized trials of nonpharmacologic treatment: explanation and elaboration. Ann Intern Med.

[51] French SD, Charity MJ, Forsdike K, Gunn JM, Polus BI, Walker BF (2013). Chiropractic observation and analysis STudy (COAST): providing an understanding of current chiropractic practice. Med J Aust.

[52] Green ME, Weir E, Hogg W, Etches V, Moore K, Hunter D (2013). Improving collaboration between public health and family health teams in Ontario. Healthc Policy.

[53] Dillman DA. Mail and internet surveys: the tailored design method: with new internet, visual, and mixed-mode guide, 2nd ed. 2007 update. Hoboken, N.J., Chichester: John Wiley & Sons, Inc., 2007.

[54] Coulter ID, Shekelle PG (2005). Chiropractic in North America: a descriptive analysis. J Manip Physiol Ther.

[55] Perreault K, Dionne CE, Rossignol M, Poitras S, Morin D (2014). Physiotherapy practice in the private sector: organizational characteristics and models. BMC Health Serv Res.

[56] Page M, French S, McKenzie J, O'Connor D, Green S (2011). Recruitment difficulties in a primary care cluster randomised trial: investigating factors contributing to general practitioners' recruitment of patients. BMC Med Res Methodol.

[57] Henschke N, Maher CG, Refshauge KM, Herbert RD, Cumming RG, Bleasel J (2008). Prognosis in patients with recent onset low back pain in Australian primary care: inception cohort study. BMJ.

[58] Mior S, Wong J, Sutton D, Beliveau P, Bussières A, French S (2016). Ontario chiropractic observation and analysis STudy (O-COAST): improving quality of care through better understanding of current chiropractic practice.

[59] Patel ND, Broderick DF, Burns J, Deshmukh TK, Fries IB, Harvey HB (2016). ACR appropriateness criteria low Back pain. J Am Coll Radiol.

[60] Downie A, Williams CM, Henschke N, Hancock MJ, Ostelo RW, de Vet HC (2013). Red flags to screen for malignancy and fracture in patients with low back pain: systematic review. BMJ.

[61] World Health Organization. International Classification of Primary Care, Second edition (ICPC-2). Geneva: World Health Organization. Available at: https://www.who.int/classifications/icd/adaptations/icpc2/en/. Accessed Jan 2019.

[62] ICPC-2 PLUS: The BEACH coding system. Available: http://sydney.edu.au/medicine/fmrc/icpc-2-plus/index.php. Accessed Jan 2019.

[63] Charity MJ, French SD, Forsdike K, Britt H, Polus B, Gunn J (2013). Extending ICPC-2 PLUS terminology to develop a classification system specific for the study of chiropractic encounters. Chiropr Man Therap.

[64] Keating JL, McKenzie JE, O'Connor DA, French S, Walker BF, Charity M, et al. Providing services for acute low-back pain: A survey of Australian physiotherapists. Man Ther. 2016;22:145-52. 10.1016/j.math.2015.11.005 26732898

[65] Walker BF, French SD, Page MJ, O'Connor DA, McKenzie JE, Beringer K (2011). Management of people with acute low-back pain: a survey of Australian chiropractors. Chiropr Man Therap.

[66] Singh J, Liddy C, Hogg W, Taljaard M (2015). Intracluster correlation coefficients for sample size calculations related to cardiovascular disease prevention and management in primary care practices. BMC Res Notes.

[67] Campbell M, Grimshaw J, Steen N (2000). Sample size calculations for cluster randomised trials. Changing professional practice in Europe group (EU BIOMED II concerted action). J. Health Serv. Res. Policy.

[68] Francis JJ, Johnston M, Robertson C, Glidewell L, Entwistle V, Eccles MP (2010). What is an adequate sample size? Operationalising data saturation for theory-based interview studies. Psychol Health.

[69] Michie S, Johnston M, Abraham C, Lawton R, Parker D, Walker A (2005). Making psychological theory useful for implementing evidence based practice: a consensus approach. Qual Saf Health Care.

[70] Cane J, O'Connor D, Michie S (2012). Validation of the theoretical domains framework for use in behaviour change and implementation research. Implement Sci.

[71] Symonds TL, Burton AK, Tillotson KM, Main CJ (1996). Do attitudes and beliefs influence work loss due to low back trouble?. Occup Med (Lond).

[72] Silverman D (2010). Qualitative Reseach: theory, method and practices.

[73] Lowe B, Wahl I, Rose M, Spitzer C, Glaesmer H, Wingenfeld K (2010). A 4-item measure of depression and anxiety: validation and standardization of the patient health Questionnaire-4 (PHQ-4) in the general population. J Affect Disord.

[74] Roland M, Fairbank J (2000). The Roland-Morris disability questionnaire and the Oswestry disability questionnaire. Spine (Phila Pa 1976).

[75] Hill JC, Dunn KM, Lewis M, Mullis R, Main CJ, Foster NE (2008). A primary care back pain screening tool: identifying patient subgroups for initial treatment. Arthritis Rheum.

[76] Hill JC, Whitehurst DG, Lewis M, Bryan S, Dunn KM, Foster NE (2011). Comparison of stratified primary care management for low Back pain with current best practice (STarT Back): a randomised controlled trial. Lancet.

[77] Miller RP, Kori S, Todd D (1991). The Tampa scale: a measure of kinesiophobia. Clin J Pain.

[78] Hirsh AT, George SZ, Riley JL, Robinson ME (2007). An evaluation of the measurement of pain catastrophizing by the coping strategies questionnaire. Eur J Pain.

[79] Walker BF, Losco B, Clarke BR, Hebert J, French S, Stomski NJ (2011). Outcomes of usual chiropractic, harm & efficacy, the OUCH study: study protocol for a randomized controlled trial. Trials.

[80] Buchbinder R, Staples M, Jolley D (2009). Doctors with a special interest in back pain have poorer knowledge about how to treat back pain. Spine.

[81] McKenzie JE, French SD, O'Connor DA, Grimshaw JM, Mortimer D, Michie S (2008). IMPLEmenting a clinical practice guideline for acute low back pain evidence-based manageMENT in general practice (IMPLEMENT): cluster randomised controlled trial study protocol. Implement Sci.

