# Supplementary material for: Imaging use for low back pain by Ontario primary care clinicians: protocol for a mixed methods study – the Back ON study
Source: BMC Musculoskelet Disord. 2019 Feb 2;20:50. doi: 10.1186/s12891-019-2427-1 (PMC6359752; doi:10.1186/s12891-019-2427-1)
Supplement: Supplementary file 1 — Data collection forms. (DOCX 331 kb) [file 12891_2019_2427_MOESM1_ESM.docx]

**Appendix 1: Practitioner Questionnaire**

1. Age
2. Are you: Male/Female/Intersex/I prefer not to disclose information concerning my sex
3. At which educational institution did you complete your chiropractic/physiotherapy/family doctor training?
4. How many years have you practised as a chiropractor/physiotherapy/family doctor?
5. Are you a member of any of the following organisations? (*different options available for each profession*)
   1. Chiropractors: Canadian Chiropractic Association (CCA); Ontario Chiropractic Association (OCA); Canadian Memorial Chiropractic College (CMCC); Canadian Chiropractic Protective Association (CCPA); Other:____________; None
   2. Physiotherapists: OPA/CPA; Orthopaedic Division of CPA; Canadian Academic of Manipulative Physiotherapy (CAMPT); Other:____________; None
   3. Family doctors: CFPC (College of Family Physicians of Canada); OCFP (Ontario College of FPs); SGFP (section of general and family practice of Ontario Medical Association); Other:____________; None
6. How many health practitioners work at this practice in total (including yourself)?

Chiropractor/physiotherapist/doctor (family)/doctor (other specialty)/occupational therapist/social worker/psychologist/massage therapist/kinesiologist/exercise specialist/nurse/nurse practitioner/Dietician/other (please specify) _________

1. Are any of the following services located/available at the same premises as your practice? (*Tick all that apply. Include services in the same building or within 50 metres, available on a daily or regular basis*):
   1. Pharmacy
   2. Laboratory tests (blood, urine, etc.)
   3. On-site imaging service (take your own x-rays/imaging)
   4. Imaging service (you refer your patients to)
   5. Other (please specify)
2. In a typical week, how many hours do you spend in clinical practice?
3. In a typical week, how many patients do you see?
4. In a typical month, how many patients would consult you with a primary complaint of low back pain?
5. How long do you typically spend with your patients?
   1. New patient visits: _____ minutes
   2. Repeat patient visits: _____ minutes
6. Do you have a special area of interest in your practice?
   1. Low back pain
   2. Chronic pain
   3. Musculoskeletal
   4. Sports medicine
   5. Other (please specify)
   6. None
7. Which clinical practice guideline/s for low back pain are you familiar with? (never heard of it/ heard of it, but have not read/likely read it at some point but can’t recall/I can recall reading it/I can recite some of the key points/I review it regularly)
   1. Stochkendahl, et al. National Clinical Guidelines for non-surgical treatment of patients with recent onset low back pain or lumbar radiculopathy. Eur Spine J 2018;27(1):60-75
   2. National Institute for Health and Care Excellence (NICE) Clinical Guidelines, 2016. Low back pain and sciatica in over 16s: assessment and management. London: National Institute for Health and Care Excellence (UK), 2016
   3. Qaseem, et al. Noninvasive Treatments for Acute, Subacute, and Chronic Low Back Pain: A Clinical Practice Guideline From the American College of Physicians. Ann Intern Med 2017;166(7):514-30
   4. Chou, et al. Diagnostic imaging for low back pain: advice for high-value health care from the American College of Physicians. Ann Intern Med 2011;154(3):181-9
   5. Other (please specify) _______________
8. Please select a response that indicates how well you agree with the following statements (Strongly Disagree/Disagree/Neither Disagree nor Agree/Agree/Strongly Agree):
   1. I do not think it is really safe for a person with low back pain to be physically active
   2. Lumbar spine imaging (x-rays, CT or MRI) are useful to confirm the diagnosis and to direct appropriate treatment of low back pain, even in the absence of red flags for serious disease
   3. There is a role for lumbar spine imaging (x-rays, CT or MRI) when there are neurological signs associated with low back pain
   4. I am likely to refer low back pain patients for lumbar spine imaging (x-rays, CT or MRI) because patients often expect me to do so
9. *(for chiropractors only)* What chiropractic technique/s or system do you mainly use?
   1. Diversified
   2. Gonstead
   3. Activator methods
   4. Thompson technique
   5. Sacrooccipital technique (SOT)
   6. Applied Kinesiology (AK)
   7. Chiropractic BioPhysics (CBP)
   8. Advanced Biostructural Correction (ABC)
   9. Other (please specify)

**Appendix 2: Checklist for practice staff to screen potential patients for eligibility**

The patient must meet all of the following inclusion criteria (i.e. a ‘YES’ must be achieved for all items):

**General criteria:**

1. They are 18 years of age or older: Y/N
2. They understand and read English: Y/N
3. They are not pregnant (females only): Y/N
4. They possess an Ontario Health Insurance Plan (OHIP) number

**Back pain criteria:**

1. This is a new episode of low back pain, defined as the current episode of pain preceded by at least 4 weeks without significant low back pain: Y/N

*Note: Patients will be included if they have a new episode, or acute exacerbation, of low back pain. This is defined as the current episode of pain, with or without leg pain, preceded by at least 4 weeks without significant low back pain. Significant low back pain is when average pain intensity is scored as 3 or more on a scale of 0 to 10, and the pain interferes with daily activities.*

1. The current episode of low back pain has been present for less than 3 months: Y/N
2. The patient’s pain is in the region bound by the lower ribs and the lower gluteal fold, with or without referred/radicular leg pain: Y/N
3. This is the first time they are seeing the participating practitioner for this episode of low back pain: Y/N

**Appendix 3: Encounter forms (initial visit and follow up visits)**


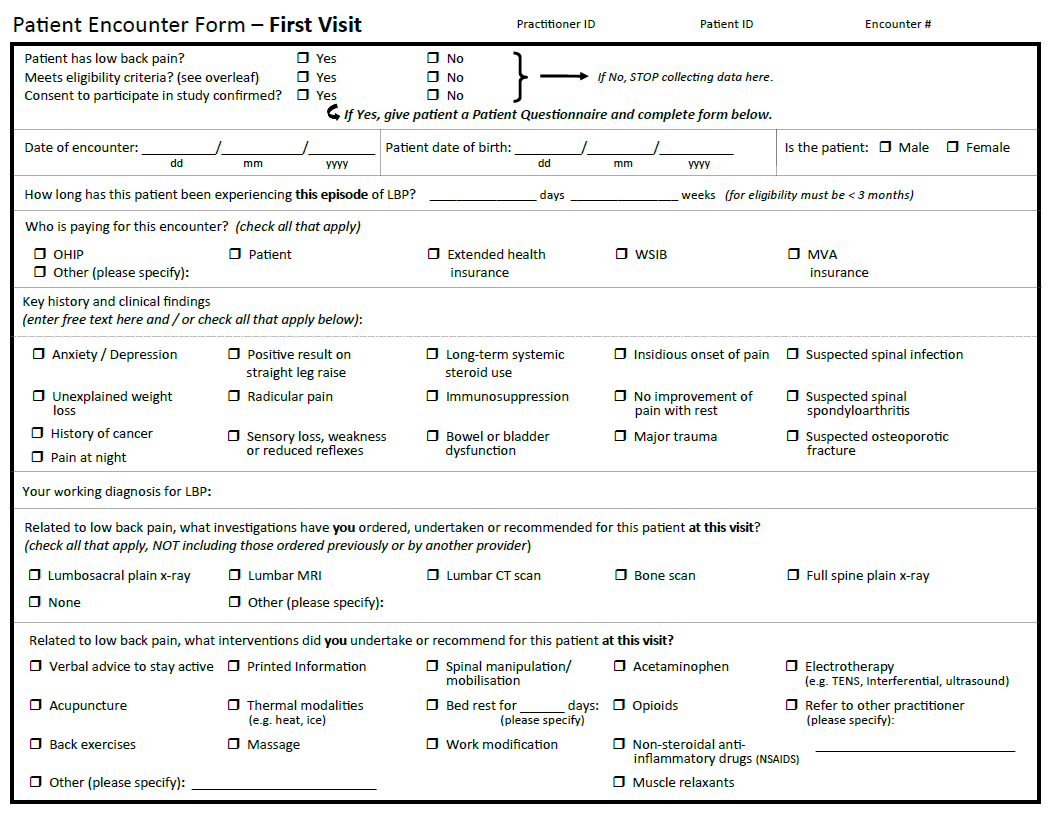


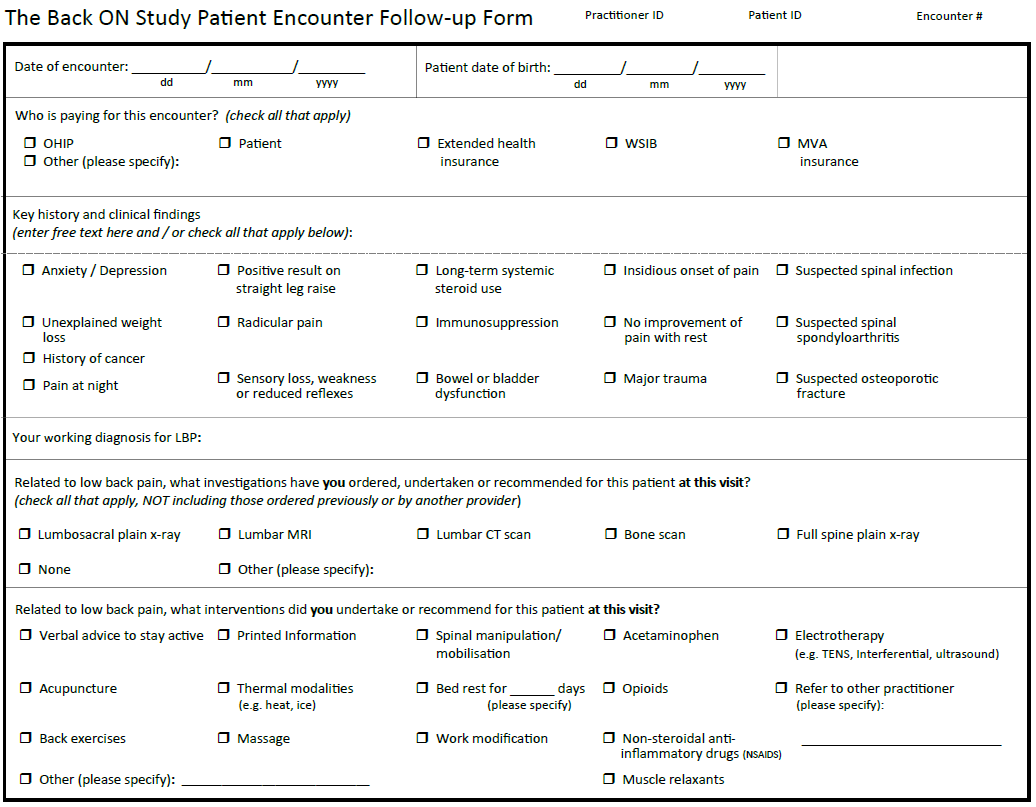


**Appendix 4: Patient Questionnaire to be completed at first visit**

*Thank you for your participation in this research project. Please fill out the following questionnaire and hand the completed form to the clinic staff.*

Today’s Date: _______ / ________ / ________

dd mm yyyy

**Some questions about your low back pain**

1. How long have you had your current episode of back pain? ________ days ________ weeks
   *Please note that many people have multiple episodes of back pain, but we specifically wish to know the duration of* ***this*** *current episode. An episode is defined as the current episode of pain preceded by at least 4 weeks without significant low back pain*
2. For the 4 weeks preceding this current episode of your low back pain, which of the following statements is true for you? *Please choose one option*

❒ Before this current episode of my low back pain, I had no low back pain for at least the last 4 weeks

❒ Before this current episode of my low back pain, I had some mild low back pain during the last 4 weeks

1. Have you had previous episodes of back pain?
   ❒ Yes ❒ No

If yes, how many episodes of back pain have you had in the last 12 months? _______

1. How would you rate your **back pain** intensity right now on a scale of 0 to 10?

| **NO PAIN** 0 | 1 | 2 | 3 | 4 | 5 | 6 | 7 | 8 | 9 | 10 **WORST POSSIBLE PAIN** |
| --- | --- | --- | --- | --- | --- | --- | --- | --- | --- | --- |

1. If your back pain has spread down your legs, how would you rate your **leg pain** intensity right now on a scale of 0 to 10?

| **NO PAIN**  0 | 1 | 2 | 3 | 4 | 5 | 6 | 7 | 8 | 9 | 10 **WORST POSSIBLE PAIN** |
| --- | --- | --- | --- | --- | --- | --- | --- | --- | --- | --- |

1. Is your current back pain episode more severe than is usual for you?

❒ Yes ❒ No

1. What tests, *if any*, were ordered or undertaken by your treating clinician **at this visit** for your current episode of back pain?

*(The list below contains tests that may or may not be relevant to you; please tick all that apply or use the ‘other’ category to describe anything not covered in the list provided)*

| ❒ Lumbosacral plain x-ray | ❒ Lumbar MRI | ❒ Bone scan |  |
| --- | --- | --- | --- |
| ❒ Full spine plain x-ray | ❒ Lumbar CT scan |  |  |
| ❒ None | ❒ Other *(please specify):*________________________________ | | |

1. How confident are you that your back pain will be completely gone or much better in 3 months? *Please circle your response where* ***0 = not confident*** *and* ***10 = very confident***

| **NOT CONFIDENT**  0 | 1 | 2 | 3 | 4 | 5 | 6 | 7 | 8 | 9 | 10 **VERY CONFIDENT** |
| --- | --- | --- | --- | --- | --- | --- | --- | --- | --- | --- |

1. What made you decide to come to this clinic today for your low back pain? (*check any reasons that apply*)

❒ For pain relief

❒ To find out what is wrong

❒ To get some imaging (e.g. x-ray, MRI, CT)

❒ To get advice

❒ To get a sick note or other related documentation

❒ To get a referral

❒ Other. Please specify ______

1. Have you been recently diagnosed (within the last 12 months) with a fracture of your spine?
   ❒ Yes ❒ No
2. Was **this episode** of your low back pain as a result of a **significant** trauma, such as a major car accident or a significant fall?
   ❒ Yes ❒ No

*If yes, please specify the type of trauma:* _____________________________________________

1. Have you ever had a low back operation?

❒ Yes ❒ No

1. Do you have insurance coverage for physiotherapy/chiropractic treatment (outside OHIP)?

❒ Yes ❒ No

1. Is your current episode of back pain covered by motor vehicle insurance or workplace injury insurance?
   ❒ Yes ❒ No

**Some questions about what treatment and tests you have had *before* your visit today**

1. Have you used any medications for this episode of back pain?

❒ Pain medication, please specify: ______________________________

❒ Muscle relaxant, please specify: ______________________________

❒ Other, please specify: ______________________________

1. Have you used any non-medication options for treatment for this back pain episode *prior* to today’s visit?

❒ Visited a chiropractor

❒ Visited a physiotherapist

❒ Visited a massage therapist

❒ Visited another health practitioner. Please specify _______

❒ Other. Please specify _______

1. Have you ***previously had*** an x-ray or any other imaging tests (e.g. CT scan, MRI, bone scan) for ***this*** episode of your back pain?
   ❒ Yes ❒ No

If yes, who referred you for, or recommended, this test?

| ❒ Family doctor | ❒ Chiropractor | ❒ Physiotherapist |  |
| --- | --- | --- | --- |
| ❒ Other *(please specify practitioner type):* __________________________________________ | | | |

**Some questions about you and your general health**

1. In general, how would you rate your health? (check one)

❒ Poor

❒ Fair

❒ Good

❒ Very good

❒ Excellent

1. How many days a week do you do 30 minutes or more of physical activity? The activity should be enough to quicken your breathing, this should not include housework or physical activity that may be part of your job. Number of days: ______________________________________
2. Do you currently have, or have you previously had, any of the following health conditions?
   *(tick all that apply)*

| ❒ Diabetes | ❒ Heart disease | ❒ High blood pressure | |
| --- | --- | --- | --- |
| ❒ Anemia | ❒ Kidney disease | ❒ Psoriasis | |
| ❒ Osteoporosis | ❒ Faecal incontinence | ❒ Sciatica | |
| ❒ Asthma | ❒ Stroke | ❒ Thyroid problems | |
| ❒ Rheumatoid arthritis | ❒ Ankylosing spondylitis | ❒ Liver disease *(e.g. hepatitis)* | |
| ❒ Depression or anxiety | ❒ Inflammatory bowel disease | ❒ Uveitis *(eye inflammation)* | |
| ❒ Urinary incontinence or urinary retention | ❒ Lung problems  *(e.g. Emphysema or COPD)* |  | |
| ❒ Numbness in the region of your buttocks and the inner surfaces of your thighs | ❒ Arthritis  *(Specify type, if known):*______________________________________________ | | |
| ❒ Other health conditions *(please specify*):___________________________________________ | | |  |
| ❒ I do not have, nor previously had, any of the above conditions | | |  |

1. a. Current height: ____________ cm OR inches (please circle units)

b. Current weight: ____________ lbs OR kg (please circle units)

1. Do you currently smoke?

❒ No

❒ Yes. If yes, how many cigarettes per day? _____ How many years have you smoked? _______

1. Do you have a history of cancer?

❒ Yes ❒ No

If yes, please specify which type of cancer: ____________________________________

1. Have you recently lost a lot of weight for no known reason, and not as a result of a deliberate diet?

❒ Yes ❒ No

1. Are you an intravenous drug user?

❒ Yes ❒ No

1. Are you currently taking, or have you taken in the past, oral steroids (including cortisone, hydrocortisone and prednisone)?

❒ Yes ❒ No

If Yes, for how long? ____ days ____ weeks ____ months _____ years

1. In the past two weeks, how often have you been bothered by the following problems: (*Check one box per statement*)

|  | Not at all | Several days | More than half the days | Nearly every day |
| --- | --- | --- | --- | --- |
| a. Little interest or pleasure in doing things | ❒ | ❒ | ❒ | ❒ |
| b. Feeling down, depressed or hopeless | ❒ | ❒ | ❒ | ❒ |
| c. Feeling nervous, anxious or on edge | ❒ | ❒ | ❒ | ❒ |
| d. Not being able to stop or control worrying | ❒ | ❒ | ❒ | ❒ |

1. How confident are you in the following statements?

*Please circle your response where* ***0 = not at all confident*** *and* ***6 = completely confident****:*

a. I can do some form of work, despite the pain (“work” includes housework, paid and unpaid work)

| **NOT CONFIDENT** 0 | 1 | 2 | 3 | 4 | 5 | 6 **COMPLETELY CONFIDENT** |
| --- | --- | --- | --- | --- | --- | --- |

b. I can live a normal lifestyle, despite the pain

| **NOT CONFIDENT** 0 | 1 | 2 | 3 | 4 | 5 | 6 **COMPLETELY CONFIDENT** |
| --- | --- | --- | --- | --- | --- | --- |

**Some questions about you**

1. What is your date of birth? _______ / ________ / _________

dd mm yyyy

1. Are you: ❒ Male ❒ Female ❒ Intersex
    ❒ I prefer not to disclose information concerning my sex
2. What is your home postal code? ________________
3. Are you (check one):

❒ Full time employed

❒ Part time employed

❒ Unemployed

❒ Student

❒ Retired

❒ Unable to work due to disability

❒ Other, please specify: __________

1. Highest level of education: (check one)

❒ Did not complete high school

❒ High school

❒ College/undergraduate

❒ Graduate degree

1. What is your ethnic or cultural origin (please list all)? ____________________________________

*For example, Canadian, English, Chinese, French, East Indian, Italian, German, Scottish, Cree, Mi'kmaq, Salish, Métis, Inuit, Filipino, Irish, Dutch, Ukrainian, Polish, Portuguese, Vietnamese, Korean, Jamaican, Greek, Iranian, Lebanese, Mexican, Somali, Colombian, etc*

1. Current living arrangement: (check one)

❒ Living alone in a house, condominium or apartment

❒ Living with someone else in a house, condominium or apartment

❒ Living in a residential facility (e.g., retirement home)

❒ Living in a care facility (e.g., nursing home)

❒ Other. Please specify: ___________

1. Please indicate whether you agree or disagree with the following statement by circling the appropriate number on the scale
   *Please circle your response where* ***1 = completely disagree*** *and* ***5 = completely agree***

a. X-rays or scans are necessary to get the best medical care for low back pain

| **COMPLETELY DISAGREE** | 1 | 2 | 3 | 4 | 5 | **COMPLETELY AGREE** |
| --- | --- | --- | --- | --- | --- | --- |

b. Everyone with low back pain should have spine imaging (e.g X-ray, CT or MRI)

| **COMPLETELY DISAGREE** | 1 | 2 | 3 | 4 | 5 | **COMPLETELY AGREE** |
| --- | --- | --- | --- | --- | --- | --- |

1. Below are some descriptions of how some people’s **back pain can change over time**, with pictures to show how their pain might go up or down. Please look at these and tick the box next to the one option that you think comes closest to how your pain has been over **the last year** prior to this episode

| a) |  | A single episode with no other major episodes of back pain |  |
| --- | --- | --- | --- |
| b) |  | A few episodes of back pain, with mostly pain-free periods in between |  |
| c) |  | Some back pain most of the time, and a few episodes of severe pain |  |
| d) |  | Pain that goes up and down all the time, with episodes of severe back pain |  |
| e) |  | Severe back pain all or nearly all of the time |  |
| f) |  | Back pain that has got gradually worse |  |
| g) |  | Back pain that has improved gradually |  |
| h) |  | No back pain, or only the odd day with mild pain |  |

1. **Roland Morris Disability Questionnaire**

*When your back hurts, you may find it difficult to do some things you normally do. This list contains some sentences that people have used to describe themselves when they have back pain. When you read them, you may find that some stand out because they describe you today. As you read the list, think of yourself today. When you read a sentence that describes you today, tick the “yes” box to the left of the sentence. If the sentence does not describe you, tick the “no” box. Remember, only tick yes if you are sure that the sentence describes you today.* ***Please clearly tick Yes or No***

| **Yes** | **No** |  |
| --- | --- | --- |
| ❒ | ❒ | I stay at home most of the day because of my back pain |
| ❒ | ❒ | I change my position frequently to allow my back to be more comfortable |
| ❒ | ❒ | I walk slower than usual because of my back pain |
| ❒ | ❒ | Because of my back pain, I am not doing any of the jobs that I usually do around the house |
| ❒ | ❒ | Because of my back pain, I use a handrail to get upstairs |
| ❒ | ❒ | Because of my back pain, I lie down to rest more often than usual |
| ❒ | ❒ | Because of my back pain, I have to hold on to something to get out of an armchair |
| ❒ | ❒ | Because of my back pain, I ask other people to do things for me |
| ❒ | ❒ | I get dressed slower than usual because of my back pain |
| ❒ | ❒ | I stand up only for short periods of time because of my back pain |
| ❒ | ❒ | Because of my back pain, I try not to bend over or kneel down |
| ❒ | ❒ | I find it difficult to get out of a straight-backed chair because of my back pain |
| ❒ | ❒ | My back is painful most of the day |
| ❒ | ❒ | I find it difficult to turn over in bed because of my back pain |
| ❒ | ❒ | Because of my back pain, my appetite is not very good |
| ❒ | ❒ | I have trouble putting on my socks (or stockings) because of my back pain |
| ❒ | ❒ | Because of my back pain, I walk only short distances |
| ❒ | ❒ | I sleep less than usual because of my back pain |
| ❒ | ❒ | Because of my back pain, I get dressed with help from someone else |
| ❒ | ❒ | I spend most of the day sitting because of my back pain |
| ❒ | ❒ | I avoid heavy jobs around the house because of my back pain |
| ❒ | ❒ | Because of my back pain, I am more irritable and bad tempered than usual with people |
| ❒ | ❒ | Because of my back pain, I go upstairs slower than usual |
| ❒ | ❒ | I stay in bed most of the day because of my back pain |

1. **STarT Back questionnaire**

*For each of the following questions, think about the last two weeks, or since the problem started (if that was within the last 2 weeks). Tick the “yes” box if any of the sentences below describe you; tick “no” if the sentence does not describe you.*

| **Yes** | **No** |  |
| --- | --- | --- |
| ❒ | ❒ | My back pain has spread down my leg(s) at some time in the last 2 weeks (or since the problem started) |
| ❒ | ❒ | I have had pain in the shoulder or neck at some time in the last 2 weeks (or since the problem started) |
| ❒ | ❒ | I only walk short distances because of my back pain |
| ❒ | ❒ | In the last 2 weeks (or since the problem started) I have dressed more slowly than usual because of my back pain |
| ❒ | ❒ | I do not think it’s really safe for a person with a condition like mine to be physically active |
| ❒ | ❒ | Worrying thoughts have been going through my mind a lot of the time |
| ❒ | ❒ | I feel that my back pain is terrible and it’s never going to get any better |
| ❒ | ❒ | In general, I have stopped enjoying all the things I usually enjoy |

Overall, how bothersome has your back pain been in the last 2 weeks or since the problem started?

| ❒ Not at all | ❒ Slightly | ❒ Moderately | ❒ Very much | ❒ Extremely |
| --- | --- | --- | --- | --- |

**Appendix 5: Patient Questionnaire to be completed at follow up visits**

Today’s Date: _______ / ________ / ________

dd mm yyyy

**Some questions about your low back pain**

1. How would you rate your **back pain** intensity right now on a scale of 0 to 10?

| **NO PAIN** 0 | 1 | 2 | 3 | 4 | 5 | 6 | 7 | 8 | 9 | 10 **WORST POSSIBLE PAIN** |
| --- | --- | --- | --- | --- | --- | --- | --- | --- | --- | --- |

1. If your back pain has spread down your legs, how would you rate your **leg pain** intensity right now on a scale of 0 to 10?

| **NO PAIN** 0 | 1 | 2 | 3 | 4 | 5 | 6 | 7 | 8 | 9 | 10 **WORST POSSIBLE PAIN** |
| --- | --- | --- | --- | --- | --- | --- | --- | --- | --- | --- |

1. What tests, *if any*, were ordered or undertaken by your treating clinician **at this visit** for your back pain?

*(The list below contains tests that may or may not be relevant to you; please tick all that apply or use the ‘other’ category to describe anything not covered in the list provided)*

| ❒ Lumbosacral plain x-ray | ❒ Lumbar MRI | ❒ Bone scan |  |
| --- | --- | --- | --- |
| ❒ Full spine plain x-ray | ❒ Lumbar CT scan |  |  |
| ❒ None | ❒ Other *(please specify):*________________________________ | | |

1. How confident are you that your back pain will be completely gone or much better in 3 months? *Please circle your response where* ***0 = not confident*** *and* ***10 = very confident***

| **NOT CONFIDENT**  0 | 1 | 2 | 3 | 4 | 5 | 6 | 7 | 8 | 9 | 10 **VERY CONFIDENT** |
| --- | --- | --- | --- | --- | --- | --- | --- | --- | --- | --- |

1. Do you agree with the following statement: “I do not think it’s really safe for a person with a condition like mine to be physically active”.

❒ Yes ❒ No

1. Do you agree with the following statement: “My pain is terrible, and I think it’s never going to get any better”.

❒ Yes ❒ No

1. What made you decide to come to this clinic today for your low back pain? (*check any reasons that apply*)

❒ For pain relief

❒ To find out what is wrong

❒ To get some imaging (e.g. x-ray, MRI, CT)

❒ To get advice

❒ To get a sick note or other related documentation

❒ To get a referral

❒ Because my practitioner asked me to return for treatment

❒ Other. Please specify ______

1. How satisfied were you with the care you received from your last visit with this practitioner?

Very dissatisfied = 0; dissatisfied = 1; no preference = 2; satisfied = 3; very satisfied = 4

1. Did you experience any new unwelcome symptoms, OR an increase of your presenting symptoms, during the first 48 hours (two days) after your last treatment by this practitioner? *This includes pain, discomfort or restrictions in daily activities (for example: walking, washing, or dressing). Please indicate how severe the new symptoms, or increase in symptoms, were (0=none; 1-3=mild; 4-6=moderate; 7-10 severe)*

| **NONE** 0 | 1 | 2 | 3 | 4 | 5 | 6 | 7 | 8 | 9 | 10 **SEVERE** |
| --- | --- | --- | --- | --- | --- | --- | --- | --- | --- | --- |

**Some questions about you and your general health**

1. In general, how would you rate your health? (check one)

❒ Poor

❒ Fair

❒ Good

❒ Very good

❒ Excellent

1. Since your ***last visit*** with this practitioner, have you been diagnosed with, or do you have, any of the following health conditions? *(tick all that apply)*

❒ Osteoporosis

❒ Sciatica

❒ Fracture in your spine

❒ Infection in your spine

❒ Cancer

❒ Arthritis. (Specify type, if known): ______________________________________________

❒ Numbness in the region of your buttocks and the inner surfaces of your thighs

❒ Other health conditions *(please specify*):___________________________________________

❒ I have not been diagnosed with any of the above health conditions since my last visit

1. Since your last visit with this practitioner how often have you been bothered by the following problems: (*Check one box per statement*)

|  | Not at all | Several days | More than half the days | Nearly every day |
| --- | --- | --- | --- | --- |
| a. Little interest or pleasure in doing things | ❒ | ❒ | ❒ | ❒ |
| b. Feeling down, depressed or hopeless | ❒ | ❒ | ❒ | ❒ |
| c. Feeling nervous, anxious or on edge | ❒ | ❒ | ❒ | ❒ |
| d. Not being able to stop or control worrying | ❒ | ❒ | ❒ | ❒ |

1. How confident are you in the following statements? *Please circle your response where* ***0 = not at all confident*** *and* ***6 = completely confident****:*

a. I can do some form of work, despite the pain (“work” includes housework, paid and unpaid work)

| **NOT CONFIDENT** 0 | 1 | 2 | 3 | 4 | 5 | 6 **COMPLETELY CONFIDENT** |
| --- | --- | --- | --- | --- | --- | --- |

b. I can live a normal lifestyle, despite the pain

| **NOT CONFIDENT** 0 | 1 | 2 | 3 | 4 | 5 | 6 **COMPLETELY CONFIDENT** |
| --- | --- | --- | --- | --- | --- | --- |

**Appendix 6: Patient 3, 6 and 12-month questionnaires**

Today’s Date: _______ / ________ / ________

1. How would you rate your **back pain** intensity right now on a scale of 0 to 10?

| **NO PAIN** 0 | 1 | 2 | 3 | 4 | 5 | 6 | 7 | 8 | 9 | 10 **WORST POSSIBLE PAIN** |
| --- | --- | --- | --- | --- | --- | --- | --- | --- | --- | --- |

1. If your back pain has spread down your legs, how would you rate your **leg pain** intensity right now on a scale of 0 to 10?

| **NO PAIN** 0 | 1 | 2 | 3 | 4 | 5 | 6 | 7 | 8 | 9 | 10 **WORST POSSIBLE PAIN** |
| --- | --- | --- | --- | --- | --- | --- | --- | --- | --- | --- |

1. In the last 3 months/6 months (<*delete whichever does not apply*>), have you been diagnosed with, or do you have, any of the following health conditions? *(tick all that apply)*

❒ Osteoporosis

❒ Sciatica

❒ Fracture in your spine

❒ Infection in your spine

❒ Cancer

❒ Arthritis. (Specify type, if known): ______________________________________________

❒ Numbness in the region of your buttocks and the inner surfaces of your thighs

❒ Other health conditions *(please specify*):___________________________________________

❒ I have not developed any of the above conditions in the last 3 months/6 months (<*delete whichever does not apply*>)

If yes to any of the above conditions, please state the approximate date of your diagnosis: ____________

1. Please indicate if you have received any of the following tests for your low back pain in the last 3 months/6 months:

❒ X-ray. If yes, who ordered this x-ray: _________

❒ MRI. If yes, who ordered this MRI: _________

❒ CT scan. If yes, who ordered this CT scan: _________

❒ Other test. Please specify which test and who ordered the test: ______________

1. Compared to when this episode first started, how would you describe your back pain these days?

| **MUCH WORSE** | -5 | -4 | -3 | -2 | -1 | 0  UNCHANGED | +1 | +2 | +3 | +4 +5 **MUCH BETTER** |
| --- | --- | --- | --- | --- | --- | --- | --- | --- | --- | --- |

**Roland Morris Disability Questionnaire**

*When your back hurts, you may find it difficult to do some things you normally do. This list contains some sentences that people have used to describe themselves when they have back pain. When you read them, you may find that some stand out because they describe you today. As you read the list, think of yourself today. When you read a sentence that describes you today, tick the “yes” box to the left of the sentence. If the sentence does not describe you, tick the “no” box. Remember, only tick yes if you are sure that the sentence describes you today.* ***Please clearly tick Yes or No***

| **Yes** | **No** |  |
| --- | --- | --- |
| ❒ | ❒ | I stay at home most of the day because of my back pain |
| ❒ | ❒ | I change my position frequently to allow my back to be more comfortable |
| ❒ | ❒ | I walk slower than usual because of my back pain |
| ❒ | ❒ | Because of my back pain, I am not doing any of the jobs that I usually do around the house |
| ❒ | ❒ | Because of my back pain, I use a handrail to get upstairs |
| ❒ | ❒ | Because of my back pain, I lie down to rest more often than usual |
| ❒ | ❒ | Because of my back pain, I have to hold on to something to get out of an armchair |
| ❒ | ❒ | Because of my back pain, I ask other people to do things for me |
| ❒ | ❒ | I get dressed slower than usual because of my back pain |
| ❒ | ❒ | I stand up only for short periods of time because of my back pain |
| ❒ | ❒ | Because of my back pain, I try not to bend over or kneel down |
| ❒ | ❒ | I find it difficult to get out of a straight-backed chair because of my back pain |
| ❒ | ❒ | My back is painful most of the day |
| ❒ | ❒ | I find it difficult to turn over in bed because of my back pain |
| ❒ | ❒ | Because of my back pain, my appetite is not very good |
| ❒ | ❒ | I have trouble putting on my socks (or stockings) because of my back pain |
| ❒ | ❒ | Because of my back pain, I walk only short distances |
| ❒ | ❒ | I sleep less than usual because of my back pain |
| ❒ | ❒ | Because of my back pain, I get dressed with help from someone else |
| ❒ | ❒ | I spend most of the day sitting because of my back pain |
| ❒ | ❒ | I avoid heavy jobs around the house because of my back pain |
| ❒ | ❒ | Because of my back pain, I am more irritable and bad tempered than usual with people |
| ❒ | ❒ | Because of my back pain, I go upstairs slower than usual |
| ❒ | ❒ | I stay in bed most of the day because of my back pain |

**STarT Back questionnaire**

*For each of the following questions, think about the last two weeks, or since the problem started (if that was within the last 2 weeks). Tick the “yes” box if any of the sentences below describe you; tick “no” if the sentence does not describe you.*

| **Yes** | **No** |  |
| --- | --- | --- |
| ❒ | ❒ | My back pain has spread down my leg(s) at some time in the last 2 weeks (or since the problem started) |
| ❒ | ❒ | I have had pain in the shoulder or neck at some time in the last 2 weeks (or since the problem started) |
| ❒ | ❒ | I only walk short distances because of my back pain |
| ❒ | ❒ | In the last 2 weeks (or since the problem started) I have dressed more slowly than usual because of my back pain |
| ❒ | ❒ | I do not think it’s really safe for a person with a condition like mine to be physically active |
| ❒ | ❒ | Worrying thoughts have been going through my mind a lot of the time |
| ❒ | ❒ | I feel that my back pain is terrible and it’s never going to get any better |
| ❒ | ❒ | In general, I have stopped enjoying all the things I usually enjoy |

Overall, how bothersome has your back pain been in the last 2 weeks or since the problem started?

| ❒ Not at all | ❒ Slightly | ❒ Moderately | ❒ Very much | ❒ Extremely |
| --- | --- | --- | --- | --- |
